# Perception of patient safety culture among perioperative staff: exploring the roles of individual factors and organizational factors

**DOI:** 10.1186/s12913-025-13531-w

**Published:** 2025-10-06

**Authors:** Yi Lin Bong, Keng Sheng Chew, Sze Kiat Sim, Shalin Wan Fei Lee, Peter Chee Seong Tan

**Affiliations:** 1 Faculty of Medicine and Health Sciences, Universiti Malaysia Sarawak, Kota Samarahan, Sarawak 94300 Malaysia; 2 Department of Anesthesiology and Intensive Care, Sarawak General Hospital, Kuching, Sarawak 93586 Malaysia

**Keywords:** Patient safety culture, Individual factors, Organizational factors, Perioperative staff, Safety attitude questionnaire-operating room (SAQ-OR)

## Abstract

**Background:**

Patient safety is a fundamental concern in healthcare, especially in high-risk settings such as operating theaters, where there is an increased likelihood of adverse events. Nonetheless, studies within the operating theater setting remain limited. This study examined the influence of organizational and individual factors, and the moderating effects of job position, gender, and experience toward patient safety culture among perioperative staff at Sarawak General Hospital (SGH) in Malaysia.

**Methods:**

A cross-sectional study design was employed, involving 137 perioperative staff members, including doctors and nurses, selected through proportionate stratified sampling. The Safety Attitude Questionnaire-Operating Room (SAQ-OR) and Hospital Survey on Patient Safety Culture (HSOPSC) was adapted in this study. The responses were analyzed via descriptive analysis and partial least square-structural equation modeling (PLS-SEM).

**Results:**

Most respondents (67.2%) were registered nurses. The stress recognition dimension had the highest mean positive response rate at 73.0%, whereas the perceptions of the management dimension had the lowest at 22.6%. The study findings revealed a significant positive relationship between individual factors and the perception of patient safety culture (β = 0.389, *p* = 0.008). In contrast, the association between organizational factors and the perception of patient safety culture was positive but not significant (β = 0.293, *p* = 0.054). The moderating effects of position (β = 0.048, *p* = 0.572), gender (β = 0.183, *p* = 0.515), and years of experience in a specialty (β = −0.103, *p* = 0.187) were also nonsignificant.

**Conclusion:**

Overall, the perception of patient safety culture in the SGH operating theater was perceived as moderate, with substantial room for improvement. The lowest positive response rate in the management dimension implies the need for the organization to address staffing shortage issues and improve workplace support to increase patient safety. Individual factors, such as job satisfaction and stress recognition, were significantly associated with these perceptions. With a coefficient of determination (R²) value of 42.6% in this structural model, additional influencing factors may be relevant. Future studies should explore influences such as government policies, budget allocation, and technological advancements to further enhance patient safety culture in operating theaters.

## Background

Defined as a state of being free from preventable harm during treatment, patient safety is a crucial element in healthcare services delivery [1]. The global spotlight on patient safety is further intensified following the publications of “To Err is Human: Building a Safer Health System” by the Institute of Medicine in the United States [2] and “An Organization with a Memory” by the United Kingdom Department of Health [3]. These reports revealed an approximate 10% error rate in hospital admissions and mortality that were attributed to potentially preventable adverse events. The World Health Organization (WHO) indicates that adverse events continue to affect about one in every ten hospitalized patients worldwide, with nearly 50% deemed preventable [4].

Adverse events, often due to miscommunication and procedural failures, can prolong hospitalization stays, incur legal costs, and undermine the reputation of healthcare institutions [5]. In some countries, approximately 15% of hospital activities and expenditures are dedicated to addressing these adverse events [6].

In Malaysia, the increasing number of incident reports in recent years further testify to the ongoing challenges in patient safety [7]. Data from 2019 shown that of the 9431 patient safety incidents were recorded in the e-Incident Reporting (e-IR) system across 140 Ministry of Health (MOH) hospitals, with surgical departments contributing 15.8% of cases [8]. The Joint Commission Sentinel Event Data 2022 Annual Review revealed that unintended retention of foreign objects and wrong-site surgeries each contributed to approximately 6% of the reported sentinel events, often due to multiple factors including failures to follow standardized procedures, communication breakdowns and poor teamwork [9].

Operating theaters (OT) are inherently high-risk environments that can present significant threats to patient safety due to their complex nature as well as human resource challenges [6]. Inadequate staff and heavy workloads can lead to stress and fatigue among perioperative staff, reducing staff’s vigilance and increasing the likelihood of errors [10]. In addition, sociotechnical, cognitive and environmental challenges which include suboptimal equipment layout, further heightens operational risks in modern surgical environments [11].

Whilst a number of past studies had explored the perceptions and attitudes toward patient safety among different categories of Malaysian healthcare staff in various settings [5, 12–17], limited studies have been conducted within the OT setting to address these critical aspects of patient safety. Local millennial healthcare professionals have been found to encounter challenges in critical dimensions such as handoff procedures, staffing levels, error reporting, and teamwork dynamics [5]. Addressing this gap is significant, as targeted understanding within perioperative contexts is essential to designing effective safety interventions.

This study aimed to evaluate the perception of patient safety culture among perioperative staff at Sarawak General Hospital (SGH). It sought to explore the relationships between organizational and individual factors and their influence on the perception of patient safety culture. Additionally, the study also examined the moderating effects of job position, gender, and experience on the relationship between individual factors and the perception of patient safety culture among perioperative staff. The study addressed the following hypotheses:

### H1

There are significant relationships between organizational factors and the perception of patient safety culture among perioperative staff.

### H2

There are significant relationships between individual factors and the perception of patient safety culture among perioperative staff.

### H3

Job position, gender, and experience moderate the relationship between individual factors and the perception of patient safety culture among perioperative staff.

## Methods

### Study design

A quantitative, cross-sectional research design was employed to assess perioperative staff perceptions and relationships between organizational, individual, and moderating variables.

### Study setting

The study was conducted in the operating theater of Sarawak General Hospital (SGH), a tertiary referral hospital in Sarawak, Malaysia. Data collection took place from May to July 2023.

### Population, sample and sampling

The study population comprised all perioperative staff working in the operating theater at SGH, including surgeons, anesthetists, medical officers, medical assistants, nurse practitioners, clinical nurse specialists, and registered nurses with more than six months of experience in the department. The exclusion criteria included medical and nursing students on short term clinical attachment, nonclinical staff, house officers and clinical staff who had fewer than 6 months’ experience in operating theatre.

The total eligible population consisted of 293 perioperative staff: 72 surgeons, 88 anesthetists (including medical officers), and 133 perioperative nurses. An a priori sample size calculation was performed using GPower software [18]. With an assumed medium effect size (f² = 0.15), α = 0.05, statistical power (1–β) = 0.95, and two predictors (organizational and individual factors), the minimum required sample was 107 respondents. To allow for an anticipated 20% non-response, the adjusted target sample was 128 participants. In total, 137 perioperative staff completed the survey, yielding a response rate of 46.8%.

### Materials

This study adapted the 30 items from the original English SAQ-OR version [19]. Additionally, 2 items from the frequency of the event-reporting dimension of the Hospital Survey on Patient Safety Culture (HSOPSC) questionnaire were adapted [20]. The 32-item questionnaire measured patient safety dimensions via a 5-point Likert scale (with scores of 1 = “strongly disagree”, 2 = “disagree”, 3 = “neutral”, 4 = “agree”, and 5 = “strongly agree”). The dimensions measured were teamwork, safety climate, job satisfaction, stress recognition, perceptions of management, working conditions, and frequency of event reporting.

Three items in the questionnaire were negatively worded and were therefore reverse-coded prior to analysis: (1) *“In the ORs here*,* it is difficult to speak up if I perceive a problem with patient care”* (teamwork), (2) *“In the OR*,* it is difficult to discuss errors”* (safety climate), and (3) *“Hospital management does not knowingly compromise the safety of patients”* (perceptions of management).

The respondents also graded their broad perceptions on individual factors, organizational factors in their organization as well as the overall patient safety perception using the scores 1 = “poor”, 2 = “fair”, 3 = “good”, 4 = “very good” and 5 = “excellent”.

### Procedures

Ethical approval was obtained from the Medical Research and Ethics Committee (MREC), Ministry of Health Malaysia (NMRR ID-22-02887-9UP). Formal permission was also obtained from the director of SGH prior to the commencement of this study. After obtaining informed consent from the participants, the self-administered questionnaires were distributed to the respondents. Participants were informed that completion of the questionnaire would require approximately 15 min, and they were encouraged to respond at their convenience given their clinical schedules. To ensure confidentiality, completed questionnaires were returned in a sealed collection box placed in the management office.

### Data analysis

All data collected were entered into Microsoft Excel and subsequently analyzed using the Statistical Package for the Social Sciences (SPSS) version 26 and SmartPLS 4.

The original SAQ stipulates ‘not applicable’ as a missing value with no score [19]. Overall, missing data accounted for only 0.08% of total responses. Because this level was minimal, missing values were imputed in SmartPLS using mean replacement. This method is appropriate when missing values are below 5% per indicator, as it preserves the sample size and avoids the bias that can result from casewise deletion [21].

Since Partial Least Squares Structural Equation Modelling (PLS-SEM) is a non-parametric method that does not assume data normality, no normality tests were required. This makes PLS-SEM appropriate for this study, which involved perceptual survey data that may deviate from multivariate normality [21].

Descriptive analysis (mean, standard deviation, frequencies, and percentages) was used to summarize socio-demographic characteristics and patient safety dimension scores. The 5-point Likert scale scores were transformed into a 100-point scale, with the following values: 1 = 0, 2 = 25, 3 = 50, 4 = 75, and 5 = 100 [17]. The mean score for each dimension was calculated by summing the scores of the items within that dimension and dividing by the number of items. Positive response rates were also determined on the basis of percentage of respondents who selected “agree” or “agree strongly” (4 or 5 on the 5-point Likert scale) for each item. A score equal to or greater than 75% indicated a positive safety attitude, whereas scores equal to or less than 50% highlighted areas requiring improvement [22].

PLS-SEM was employed as it has been used to predict and explain variance in safety culture research [23]. The PLS-SEM approach can simultaneously analyze multiple dependent and independent variables, allowing for a more comprehensive examination of complex relationships [21].

The two-stage approach was used. Stage 1 involved confirmatory factor analysis (CFA) via measurement model assessment to assess the quality of the constructs. Composite reliability (CR) values > 0.7 were considered acceptable. Convergent validity was evaluated via item factor loadings and average variance extracted (AVE). Factor loadings > 0.7 were accepted, whereas factor loadings < 0.4 were removed [21]. For items with factor loadings between 0.4 and 0.7, AVE values > 0.5 were deemed acceptable [21]. Discriminant validity was confirmed via the Fornell and Larcker criterion, where the square root of the AVE for each construct exceeded its correlation with other constructs [21]. Common method variance (CMV) was assessed via Harman’s one-factor test to identify variance from measurement errors instead of actual constructs.

Stage 2 involved structural model assessment for hypothesis testing. Bootstrapping was performed with 500 resamples to evaluate the significance of path coefficients for both direct and indirect relationships. The predictive accuracy of the model was indicated by coefficient of determination (R²) values, with cutoffs of 0.26, 0.13, and 0.02 representing substantial, moderate, and weak levels of predictive accuracy, respectively [24]. The effect size (f²) measures the impact of removing an exogenous construct, with values of 0.35, 0.15, and 0.02 indicating high, medium, and small impacts, respectively [24]. The predictive relevance (Q²) was determined via a blindfolding procedure, with Q² values greater than 0 indicating the predictive relevance of the path model for that specific construct [21].

### Ethical considerations

This study was approved by the Medical Research and Ethics Committee, Ministry of Health Malaysia (NMRR ID-22-02887-9UP). Formal permission was also obtained from the director of SGH prior to the commencement of this study. Written informed consent was obtained from all participants before data collection. Participants were reassured of the confidentiality of their responses and informed of their right to withdraw from the study at any stage without penalty.

## Results

As shown in Table 1, the respondents consisted of surgeons (6.6%), anesthetists (9.5%), medical officers (9.5%), assistant medical officers (1.5%), and nurse practitioners and clinical nurse specialists (5.8%). Most of the respondents were registered nurses (67.2%). In terms of experience, 35.8% of the respondents had at least 6 to 10 years of hospital experience, whereas 43.1% had 11 years or more. Similarly, 35.8% had 6 to 10 years in their specialty, and 32.8% had 11 or more years.

**Table 1:** Demographic profile of respondents

Demographics	Frequency (*N*)	Percentage (%)
**Position**		
Surgeon	9	6.6
Anesthetist	13	9.5
Medical Officers include Postgraduate Student	13	9.5
Assistant Medical Officer	2	1.5
Nurse Practitioner, Clinical Nurse Specialist	8	5.8
Registered Nurse	92	67.2
**Gender**		
Male	28	20.4
Female	109	79.6
**Years in Hospital**		
Less than 1 year	9	6.6
1 to 5 years	20	14.6
6 to 10 years	49	35.8
11 or more years	59	43.1
**Years in Specialty**		
Less than 1 year	15	10.9
1 to 5 years	28	20.4
6 to 10 years	49	35.8
11 or more years	45	32.8

Table 2 shows the mean scores (with standard deviations) and positive response rates for each safety dimension. The highest mean score was for ‘stress recognition’ at 76.1 (SD = 25.0), whereas ‘perceptions of management’ had the lowest at 45.6 (SD = 24.3).

**Table 2:** Mean scores and positive response rates of each safety dimension

Factors	Dimensions	Mean (SD)	Average Positive Response Rate (%)
Individual	Stress Recognition	76.1 (25.0)	73.0
	Job Satisfaction	65.8 (21.2)	55.0
Organizational	Teamwork Climate	66.9 (22.5)	58.7
	Frequency of Event Reporting	64.0 (21.7)	51.1
	Working Conditions	61.5 (20.9)	**46.4**
	Safety Climate	67.1 (21.0)	61.1
	Perceptions of Management	45.6 (24.3)	**22.6**

Note: A dimension with a score ≥ 75% was considered a positive safety attitude, whereas a dimension with a score ≤ 50% was considered to need further improvement according to Zhao et al. (2019) [22]

### Stage 1 measurement model of PLS-SEM

All constructs in this model had demonstrated good construct reliability as indicated by CR > 0.7. Two items (SR2 and TC2) were iteratively removed from the analysis because of low factor loadings (< 0.40). Two other items (PM2, SC5) were iteratively removed because of their poor AVE values. After these items were removed, the remaining items had factor loadings between 0.503 and 0.942. Discriminant validity was also established through the Fornell and Larcker criterion. The results of Harman’s test indicated that the first construct accounted for only 30.182% of the overall variance. This finding suggests that CMV did not significantly impact the study results [25].

### Stage 2 structural model of PLS-SEM

**Fig. 1:**
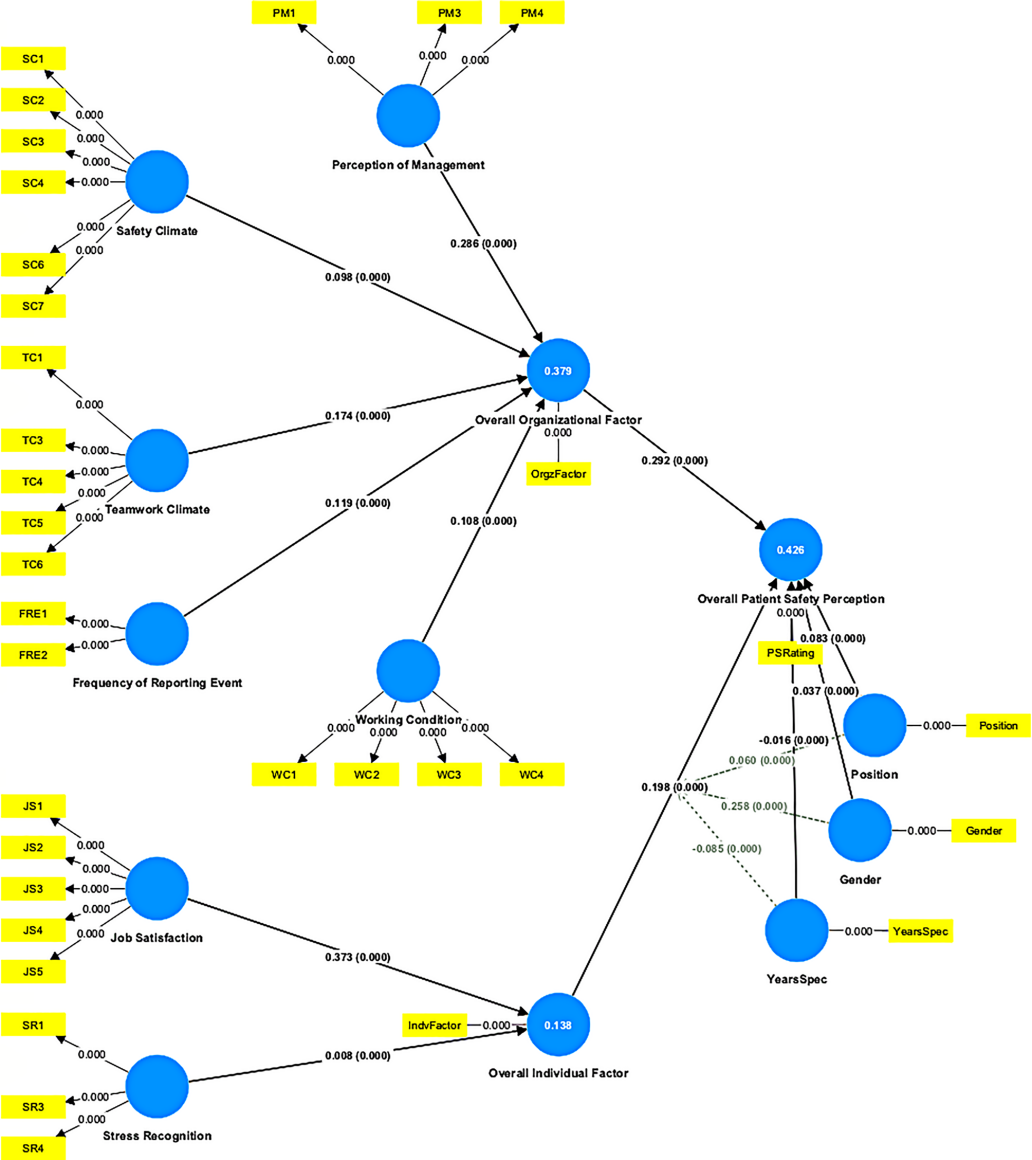
Structural model with moderating effects

As shown in Table 3, a significant positive association was found between individual factors and the perception of patient safety culture (β = 0.389, t = 2.662, *p* = 0.008). Organizational factors were positively associated with the perception of patient safety culture, though the relationship was not statistically significant at the 95% confidence interval (β = 0.293, t = 1.930, *p* = 0.054). No significant moderation effects were observed for position (β = 0.048, t = 0.565, *p* = 0.572), gender (β = 0.183, t = 0.652, *p* = 0.515), or years of experience in a specialty (β = − 0.103, t = 1.319, *p* = 0.187) on the relationship between individual factors and the perception of patient safety culture among perioperative staff (see Fig. 1).

**Table 3:** Direct hypothesis testing

Path	Original sample	Bootstrapping mean	STDEV	T	*P* value	Decision
H2: Organizational Factor -> Patient Safety Rating	0.293	0.305	0.152	1.930	0.054	Not supported
H3: Individual Factor -> Patient Safety Rating	0.389	0.380	0.146	2.662	0.008	Supported
H4: Individual Factor *Position -> Patient Safety Rating	0.048	0.040	0.086	0.565	0.572	Not supported
H5: Individual Factor *Gender -> Patient Safety Rating	0.183	0.182	0.281	0.652	0.515	Not supported
H6: Individual Factor *Years in Specialty -> Patient Safety Rating	-0.103	-0.096	0.078	1.319	0.187	Not supported

Table 4 shows the R² for individual factors was 13.8%, indicating moderate predictive accuracy, whereas the R² for organizational factors was 37.9%, indicating substantial predictive accuracy. Both factors accounted for 42.6% of the variance in the perception of patient safety culture among the perioperative staff. The Q^2^ value for the endogenous latent variables was greater than 0, confirming predictive relevance was established.

**Table 4:** Variance explained (R²) and predictive relevance (Q²)

Endogenous Variable	*R*²	Q²
Individual Factors	0.138	0.076
Organizational Factors	0.379	0.317
Perception of Patient Safety	0.426	0.286

As shown in Table 5, the effect sizes for individual and organizational factors are 0.122 and 0.068, respectively, which are small. The effect sizes for the moderating variables (position, gender, and years of experience in specialty) on the relationship between individual factors and perceptions of patient safety culture were 0.005, 0.008, and 0.017, respectively, which are considered as negligible.

**Table 5:** Effect size (f²) for main predictors

Predictor Variable	f²	Effect Size Interpretation
Individual Factors	0.122	Small
Organizational Factors	0.068	Small

## Discussion

Only 55.5% of the perioperative staff at SGH considered patient safety culture to be good and only a mere 26.3% rated their workplace as excellent or very good. This relatively low proportion suggests that overall perceptions of patient safety culture remain moderate. This finding could be attributed to the perception that due to hurried work environment and time pressures, which might result in bypassing standard operating procedures or overlooking details, thus, increasing medical error risk. This issue is likely a consequence of high patient caseloads and insufficient staffing levels [17].

In this study, perceptions of management received the lowest number of positive response rate suggesting inadequate management support and the need for better safety protocols. Working conditions also scored low in the number of positive responses, particularly in conflict-resolution processes, which is consistent with the findings from previous studies [17, 26]. The moderate reporting levels identified in this study, could likely due to an ineffective reporting system, insufficient feedback, and notably fear of disciplinary action and steep power distance index (PDI) [27]. According to Hofstede, the steep PDI in Malaysia coupled with low levels of psychological safety [28] might have discouraged junior doctors and nurses from challenging decisions from senior staff, leading to lower reporting rates [28–30].

Similarly, the safety climate and teamwork climate also scored only moderately high, suggesting a need for improved teamwork and communication. Importantly, stress recognition scored the highest, reflecting the strong awareness of the negative impact of stress and fatigue on work performance by the staff. Indeed, addressing burnout and poor mental health is crucial, as these influences can lead to increased absenteeism and staff turnover rates [31], which in turn, negatively impact team dynamics. A high turnover rate in turn, can disrupt the natural growth progression of team development as outlined in Tuckman’s model, thus, negatively impacting staff morale and productivity [32].

A significant relationship was observed in this study between individual factors (such as stress recognition and job satisfaction) and the perception of patient safety culture. This highlights the role of personal well-being in shaping safety-related behaviors. Herzberg’s two-factor theory helps explain this finding, as both hygiene factors such as supervision and workload, together with motivational factors such as recognition and growth opportunities, directly influence staff satisfaction [33]. Healthcare staff members who are satisfied are more likely to exhibit positive patient safety behaviors, thereby improving patient safety culture [17].

Similarly, recognizing and managing stress among perioperative staff is crucial for improving job satisfaction and patient safety [34, 35]. Stressful conditions such as extended on-call duties and short inter-shift durations in OT can exacerbate fatigue, and inadequate rest results in compromised work performance [36]. Similarly, local studies in the Malaysian context had also highlighted the importance of addressing burnout and mental strain among healthcare staff [31, 37].

However, perhaps a more nuanced finding from this study is that individual factors, but not organizational factors, showed significant associations with perception of patient safety culture. While this suggests that individual factors may have a particularly important influence, it is important to interpret these findings cautiously, as causality cannot be inferred from the cross-sectional design. The results instead highlight that both individual and organizational domains should be considered in parallel. This is consistent with a systematic review on the association between professional burnout and engagement with patient safety culture that revealed that although nurturing an organizational patient safety culture is important to improve staff well-being and for building a sustainable, healthy, and resilient workforce, implementing simpler measures at the individual level, such as addressing staff burnout and improving the degree of engagement and motivation in the workplace, is equally important [38]. This could also mean that even in operating theater environments with less-than-ideal organizational support, individuals who possess strong personal qualities can still maintain a positive perception of patient safety. These staff may be more resilient and adaptable, able to maintain high standards of patient safety despite challenges in their work environment, and thus, able to mitigate some of the negative impacts of a suboptimal work environment such as during the COVID-19 pandemic [39].

From a practical standpoint, these findings have implications for healthcare management. Strengthening perceptions of management may involve improving transparency, involving staff in decision-making, and providing timely feedback after incident reports. Continuous training, participation in quality improvement projects, and engagement with initiatives such as World Patient Safety Day can raise staff awareness of safety practices. Addressing stress and burnout requires structured interventions, including counselling, stress management programmes, debriefing sessions, and adequate rest between shifts, supported by strategic workforce planning to ensure manageable workloads. Fostering open communication, mentorship, and leadership walkarounds can build trust, while leadership development programmes equip managers to champion safety measures. Finally, promoting a just culture with user-friendly reporting systems and clear feedback mechanisms can encourage staff to report adverse events and contribute to safer practices.

The R² of the structural model in this study was only 42.6%, indicating that the combination of individual and organizational factors together explained less than half of the total variance in perceptions of patient safety culture. This suggests that other influencing factors might have been overlooked. This finding also implies that a reductionistic approach to categorizing influences on patient safety culture into 2 mere groups, i.e., individual and organizational factors, may be overly simplistic. In this context, to comprehensively understand the factors influencing patient safety culture, applying other frameworks such as a commonly used management tool known as the PESTLE framework (where P = Political, E = Economic, S = Social, T = Technological, L = Legal, and E = Environmental factors) may better capture a broader range of influences on patient safety culture beyond individual and organizational factors. In this regard, political factors such as the availability of government policies can directly shape institutional priorities. Economic factors, including budget allocation and financial resources, influence staffing and equipment availability. Social factors, such as cultural norms and patient expectations, may shape attitudes towards reporting and compliance. Technological factors, including the adoption of artificial intelligence or advanced monitoring tools, can help reduce staff workload and minimize human error. Legal factors, encompassing healthcare laws and regulations, may also drive safety behaviors among staff. Finally, environmental considerations, including workplace ergonomics and sustainability practices, can affect both staff performance and patient outcomes.

This study has several limitations worth noting. First, the study population was limited to one hospital in Malaysia, which may restrict its generalizability. Second, only a relatively small proportion of staff rated as “very good” or “excellent”, which suggests the results should be interpreted with caution when considering overall safety culture perceptions. Third, reliance on self-reported data may introduce social desirability bias, since participants may report more positive views instead of their real practices. Moreover, perceptions of safety culture are dynamic and could fluctuate over time or in response to specific events and daily activities. Finally, this was a quantitative study conducted via a survey questionnaire without a component of qualitative data analysis for more detailed and rich insights. Future research should integrate qualitative methods with quantitative surveys to better understand perioperative staff experiences and safety culture factors. In addition, longitudinal or intervention-based studies are recommended to explore causal relationships and to track how safety culture evolves over time and under targeted improvement strategies.

## Conclusion

In conclusion, the perception of patient safety culture among perioperative staff at SGH was perceived as moderate, with only 55.5% rating it as good and 26.3% as excellent or very good. Stress recognition was the highest-rated dimension, whereas perceptions of management received the lowest ratings, indicating significant scope for improvement. Both individual factors, such as job satisfaction and stress recognition, and organizational factors influence staff perceptions, with individual factors appearing to have a stronger association. However, the structural model explained only 42.6% of the variance, indicating that additional factors might have been overlooked. Therefore, improving patient safety requires systemic and multilevel approaches that integrate staff well-being, organizational support, and policy-level initiatives to foster a safer and more resilient healthcare environment.

## Data Availability

The data used may be obtained by contacting the corresponding author upon reasonable request.

## Abbreviations

AVE: Average Variance Extracted
CFA: Confirmatory Factor Analysis
CMV: Common Method Variance
CR: Composite Reliability
HSOPSC: Hospital Survey on Patient Safety Culture
OECD: Organization for Economic Co-operation and Development
OT: Operating Theaters
PDI: Power Distance Index
PESTLE: Political, Economic, Social, Technological, Legal, Environmental
PLS-SEM: Partial Least Square-Structural Equation Modeling
PM: Perception of Management
SAQ-OR: Safety Attitude Questionnaire-Operating Room
SGH: Sarawak General Hospital
SPSS: Statistical Package for the Social Sciences
SR: Stress Recognition
SC: Safety Climate
TC: Teamwork Climate
WHO: World Health Organization

## References

[1] Ministry of Health Malaysia. Patient Safety Council of Malaysia & Patient Safety Unit. 2024. https://patientsafety.moh.gov.my/v2/. Accessed 10 Jan 2022.

[2] Kohn LT, Corrigan JM, Donaldson MS. To err is human: Building a safer health system. Washington, DC: National Academy; 2000. 25077248

[3] Department of Health. An organization with a memory. 2000. https://webarchive.nationalarchives.gov.uk/ukgwa/20130105105027/http://www.dh.gov.uk/en/Publicationsandstatistics/Publications/PublicationsPolicyAndGuidance/DH_4065083. Accessed 16 May 2022.

[4] World Health Organization. Patient safety. 2023. https://www.who.int/news-room/fact-sheets/detail/patient-safety. Accessed 12 August 2025.

[5] Perjit S. Patient safety culture post covid-19 pandemic: in perspective of millennials human capital issues. Open J Nurs. 2021;11:1015–30. 10.4236/ojn.2021.1111081.

[6] World Health Organization. Patient safety - Facts in pictures. 2019. https://www.who.int/news-room/fact-sheets/detail/patient-safety. Accessed 20 May 2023.

[7] Alifah Z. Cases on medical negligence on the rise, Health Ministry’s data show. The Malaysian Reserve. 2019. https://themalaysianreserve.com/2019/09/18/cases-on-medical-negligence-on-the-rise-health-ministrys-data-shows/. Accessed 10 Feb 2022.

[8] Khalid KH, Yamamoto E, Hamajima N, Kariya T. Rates and factors associated with serious outcomes of patient safety incidents in malaysia: an observational study. Global J Qual Saf Healthc. 2022;5(2):31–8. 10.36401/jqsh-21-19. 10.36401/JQSH-21-19 PMC10229002 37260835

[9] The Joint Commission. Sentinel event data 2022 annual review. 2023. https://www.jointcommission.org/resources/sentinel-event/sentinel-event-data-summary/. Accessed 20 May 2023.

[10] Ackah VA, Kwashie AA. Exploring the sources of stress among operating theatre nurses in a Ghanaian teaching hospital. Int J Afr Nurs Sci. 2023;18:100540. 10.1016/j.ijans.2023.100540.

[11] Valla V, Koukoura A, Lewis A, Dahlerup B, Tsianos GI, Vassiliadis E. The impact of human factors on the safety of operating rooms and everyday surgical practice. J Adv Res Med Sci Technol. 2020;7(1):8–16. 10.24321/2394.6539.202002.

[12] Srima ES, Lua PL, Mathumalar LF. Safety culture perceptions of pharmacists in Malaysian hospitals and health clinics: a multicentre assessment using the safety attitudes questionnaire. BMJ Open. 2015;5:e008889. 10.1136/bmjopen-2015-008889. 10.1136/bmjopen-2015-008889 PMC4663412 26610761

[13] Nuaim Z, Ibrahim AT, Niza S, Muhammad Lokman MI. Patient safety culture attitudes among different healthcare professionals in selected general and district hospitals: a preliminary study. Hum Factors Ergon J. 2018;3(2):30–4. http://hfej.hfem.org/wp-content/uploads/2018/12/HFEJ-5.pdf.

[14] Kim ARJ, Chin ZH, Sharlyn P, Priscilla B, Josephine S. Hospital survey on patient safety culture in Sarawak general hospital: a cross sectional study. Med J Malaysia. 2019;74(5):385–88. 31649213

[15] Nurumal MS, Najwatul MS, Siti Hazariah AH, Muhammad Kamil CH. Nurses’ awareness on patient safety culture in a newly established university hospital. Jurnal Keperawatan Indonesia. 2020;23(2):119–27. 10.7454/jki.v23i2.1088.

[16] Salizar ML, Nur Ainun AB. Nurses’ perception on patient safety culture in critical care area at a tertiary hospital in Pahang, Malaysia. Malays J Nurs. 2020;11(4):78–84. 10.31674/mjn.2020.v11i04.008

[17] Aniza I, Siti NMK. Patient safety culture and its determinants among healthcare professionals at a cluster hospital in malaysia: a cross-sectional study. BMJ Open. 2022;12(8):e060546. 10.1136/bmjopen-2021-060546. 10.1136/bmjopen-2021-060546 PMC9403112 35995542

[18] Faul F, Erdfelder E, Lang A-G, Buchner A. G*Power 3: A flexible statistical power analysis program for the social, behavioral, and biomedical sciences. Behav Res Methods. 2007;39(2):175–91. 10.3758/BF03193146. 17695343 10.3758/bf03193146

[19] Nilsson U, Goras C, Wallentin FY, Ehrenberg A, Unbeck M. The Swedish safety attitudes Questionnaire-operating room version: psychometric properties in the surgical team. J Perianesth Nurs. 2018;33(6):935–45. 10.1016/j.jopan.2017.09.009. 30449442 10.1016/j.jopan.2017.09.009

[20] Agency for Healthcare Research and Quality. SOPS Hospital Survey. https://www.ahrq.gov/sops/surveys/hospital/index.html. Accessed 17 Jun 2022.

[21] Hair JF, Hult GTM, Ringle CM, Sarstedt MA. Primer on partial least squares structural equation modeling (PLS-SEM). 2nd ed. Thousand Oaks, CA: Sage; 2017.

[22] Zhao C, Chang Q, Zhang X, Wu QJ, Wu N, He J, Zhao YH. Evaluation of safety attitudes of hospitals and the effects of demographic factors on safety attitudes: a psychometric validation of the safety attitudes and safety climate questionnaire. BMC Health Serv Res. 2019;19:836. 10.1186/s12913-019-4682-0. 31727062 10.1186/s12913-019-4682-0 PMC6854737

[23] Tappura S, Jaaskelainen A, Pirhonen J. Creation of satisfactory safety culture by developing its key dimensions. Saf Sci. 2022;154:105849. 10.1016/j.ssci.2022.105849.

[24] Cohen J. Statistical power analysis for the behavioral sciences. 2nd ed. Hillsdale, NJ: Lawrence Erlbaum Associates; 1988.

[25] Podsakoff PM, Organ DW. Self-reports in organizational research: problems and prospects. J Manag. 1986;12(4):531–44. 10.1177/014920638601200408.

[26] Kim ARJ, Tan SH, Kho IKY, Cuki F, Lee YF, Ngian HU. Safety culture evaluation in Sarawak general hospital: a cross-sectional study using the safety attitude questionnaire (SAQ). Research Square [Preprint]. 2024. 10.21203/rs.3.rs-4801909/v1 10.2147/RMHP.S530072 PMC12399097 40895828

[27] Kaware MS, Ibrahim MI, Shafei MN, Mohd Hairon S, Abdullahi AU. Patient safety culture and its associated factors: a situational analysis among nurses in Katsina public hospitals, Northwest Nigeria. Int J Environ Res Public Health. 2022;19(6):3305. 10.3390/ijerph19063305. 35328993 10.3390/ijerph19063305 PMC8951849

[28] Kim ARJ, Nishino K, Mohamad AB, Zubalqiah Z, Inthaphatha S, Yamamoto E. What causes less speaking up for patient safety among healthcare workers? – a cross sectional study in Malaysia. Hum Resour Health. 2024;22(35). 10.1186/s12960-024-00916-x. 10.1186/s12960-024-00916-x PMC11134733 38807123

[29] Pattni N, Arzola C, Malavade A, Varmani S, Krimus L, Friedman Z. Challenging authority and speaking up in the operating room environment: a narrative synthesis. Br J Anaesth. 2019;122(2):233–44. 10.1016/j.bja.2018.10.056. 30686309 10.1016/j.bja.2018.10.056

[30] Chew KS. Applying the Art of war in the battle for patient safety. Malaysia: UNIMAS; 2024.

[31] Nursyahda Z, Nor Haniza Z, Muhammad Nur Amir AR, Lee KY. Burnout and coping strategies among nurses in malaysia: a national-level cross-sectional study. BMJ Open. 2022;12(10):e064687. 10.1136/bmjopen-2022-064687. 10.1136/bmjopen-2022-064687 PMC9557773 36216421

[32] Sanjiv K, Vaishali D, Vivek SA. Building and leading teams. Indian J Community Med. 2014;39(4):208–13. 10.4103/0970-0218.143020. 25364143 10.4103/0970-0218.143020 PMC4215500

[33] Aisyahton S, Zamzaliza AM, Siti KAS, Wan Hartini WZ. Factors contributed to job satisfaction among nurses working at tertiary hospitals in the Klang valley: an adaptation of the hertzberg’s theory. J Sustain Sci Manage. 2023;18(6):135–48. 10.46754/jssm.2023.06.012.

[34] Sonoda Y, Onozuka D, Hagihara A. Factors related to teamwork performance and stress of operating room nurses. J Nurs Manag. 2018;26(1):66–73. 10.1111/jonm.12522. 28744975 10.1111/jonm.12522

[35] Sovold LE, Naslund JA, Kousoulis AA, Saxena S, Qoronfleh MW, Grobler C, Munter L. Prioritizing the mental health and well-being of healthcare workers: an urgent global public health priority. Front Public Health. 2021;9:679397. 10.3389/fpubh.2021.679397. 34026720 10.3389/fpubh.2021.679397 PMC8137852

[36] Mohd Fadhli MF, Hanizah MY, Nur Adibah MS, Rosnawati MR, Mohd Rizal AM, Maisarah G. Fatigue and recovery among Malaysian doctors: the role of work-related activities during non-work time. BMJ Open. 2020;10(9):e036849. 10.1136/bmjopen-2020-036849. 10.1136/bmjopen-2020-036849 PMC7520834 32978189

[37] Yukthamarani P, Abdullah AM, Naresh KS, Roselina AS, Naeem H. Predicting nurses burnout through quality of work life and psychological empowerment: a study towards sustainable healthcare services in Malaysia. Sustainability. 2020;12(1):388. 10.3390/su12010388.

[38] Mossburg SE, Himmelfarb CD. The association between professional burnout and engagement with patient safety culture and outcomes: a systematic review. J Patient Saf. 2021;17(8):e1307–19. 10.1097/pts.0000000000000519. 29944601 10.1097/PTS.0000000000000519

[39] Pacutova V, Geckova AM, de Winter AF, Reijneveld SA. Opportunities to strengthen resilience of health care workers regarding patient safety. BMC Health Serv Res. 2023;23:1127. 10.1186/s12913-023-10054-0. 37858175 10.1186/s12913-023-10054-0 PMC10588085

